# Characterizing effects of hydrogen ingress in Ti–Mg based hybrid implant materials

**DOI:** 10.1039/d4ra08586h

**Published:** 2025-02-10

**Authors:** Richi Kumar, Cecilia Solís, Pavel Trtik, Armin Kriele, Wolfgang Limberg, D. C. Florian Wieland, Julian Moosmann, Maria Serdechnova, Carsten Blawert, Thomas Ebel, Regine Willumeit-Römer, Vasil M. Garamus

**Affiliations:** a German Engineering Materials Science Centre (GEMS) at Heinz Maier-Leibnitz Zentrum (MLZ), Helmholtz-Zentrum Hereon Lichtenbergstr. 1 85748 Garching Germany richi.kumar@hereon.de; b Paul Scherrer Institute 5232 Villigen Switzerland; c Institute of Metallic Biomaterials, Helmholtz Zentrum Hereon Max-Planck Str. 1 21502 Geesthacht Germany vasyl.haramus@hereon.de; d Institute of Material Physics, Helmholtz Zentrum Hereon Max-Planck Str. 1 21502 Geesthacht Germany; e Institute of Surface Science, Helmholtz Zentrum Hereon Max-Planck Str. 1 21502 Geesthacht Germany

## Abstract

Hybrid implants consisting of a permanent Ti-based part combined with a degradable Mg part, are promising solutions to design superior implants by combining the advantages of both materials. In these implants Ti provides high strength while a degradable Mg part is used for temporary structural support, bone growth stimulation or drug delivery purpose. As Mg degrades hydrogen gas is released which can ingress into the Ti part, leading to changes in its properties. The profile of hydrogen distribution is a critical parameter for mechanical stability of Ti, especially in long-term applications. To investigate this in microscopic length scale, Ti6Al4V–Mg0.6Zn0.5Ca hybrid samples prepared using metal injection molding were subjected to saline degradation for a period of 0 to 120 hours. Neutron tomography, synchrotron X-ray tomography and diffraction, SEM and gas fusion technique were used to study the ingress of hydrogen in 3D after the degradation of MgZnCa. A uniform distribution of hydrogen was seen radially while the profile along height matched with macroscopic measurements. Synchrotron XRD confirmed that the room temperature diffusion of hydrogen led to lattice expansion of the BCC *β*-phase in Ti6Al4V, while no hydride phases were present.

## Abstract 2

Hybrid implants consisting of a permanent Ti-based part combined with a degradable Mg part, are promising solutions to design superior implants by combining the advantages of both materials.

## Graphical abstract

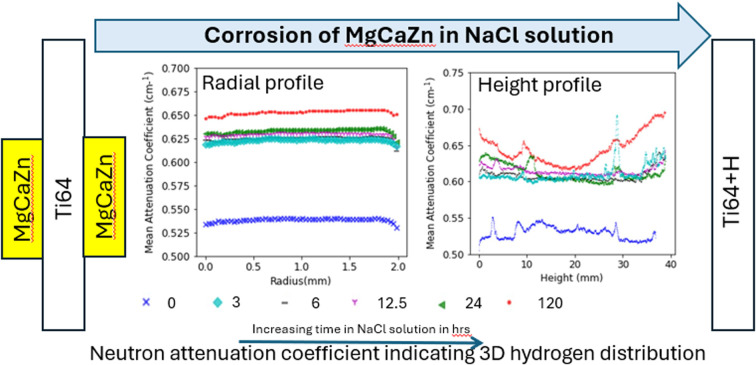

## Introduction

The demand for metallic implants has been driven by the need for fixing bone fractures, with the first successful surgery recorded in 1860s.^1^ Some of the common metals that have been investigated and used for such applications include Co-based alloys, stainless steel, Ti alloys, Mg alloys and Ni–Ti shape memory alloys.^2^ Among these Ti alloys stand out due to their low density, low elastic modulus, high specific strength, biocompatibility and corrosion resistance.^3^ They are commonly used as pacemaker cases, dental implants, maxillofacial and craniofacial implants, screws and staples for surgery, surgical instruments and components in high-speed blood centrifuges to name a few.^4^ While Ti alloys are used as permanent implants, Mg alloys have gained usage as material for temporary implants which degrade in the body and promote bone formation.^5^ This supports bone regeneration and eliminates the need for a second surgery to remove the implant.

A new avenue being explored in the field of biological implants is designing Ti–Mg hybrid implants that combine the advantages of both materials to create superior implants. In such implants Ti is used for its high strength while the Mg part dissolves over time and helps in bone stimulation.^6^ Bioactive Ti–Mg implants were developed using additive manufacturing and the hybrid parts show superior mechanical properties and adequate cell viability response.^7^ It has also been reported that compounds of Mg as a surface coating on Ti dental implants result in improved osteointegration.^8^ Titanium implants coated with thin films of mesoporous TiO_2_ and filled with Mg showed a local release of Mg from the implant surfaces and enhanced implant retention at the early stage of healing.^9,10^ Improved healing and better bio mechanical strength in the Tibia of rabbits was noted using a Ti–Mg implant when compared to pure Ti implants.^11^

In the presence of Mg in close contact with Ti, a Mg–Ti galvanic cell is formed due to a difference in standard reduction potential.^12^ Ti acts as a cathode and Mg as an anode and the galvanic coupling causes an accelerated galvanic corrosion of Mg due to the enhanced rate of oxidation and reduction reactions.^13^

While several studies have confirmed improved biological behaviour, it is also important to investigate how the dissolution of Mg impacts the remaining Ti part. A study aimed at investigating how the presence of a Ti implant effected the corrosion of a near Mg implant found that co-implantation of Mg and Ti screws resulted in faster corrosion of Mg screws. It was suggested that a certain distance between the two must be maintained to keep the acceptable degradation rate of Mg.^14^

A previous study on hybrid Ti6Al4V–Mg0.6Ca samples by our group showed brittle failure of the Ti6Al4V part after Mg dissolution.^15^ Commercial purity Ti–Mg samples were similarly investigated and an ingress of hydrogen into the Ti was confirmed by chemical tests. XRD also showed a presence of TiH_2_ in these samples, based on which Ti-hydride were suggested to be the reason for brittle failure.

While the presence of hydrogen was confirmed, the microscopic distribution of hydrogen within the sample is not known. Such a characterisation will provide insights into how the corrosion of one part effects the chemistry and microstructure of the remaining part of hybrid metallic implants, enabling better design of such implants. Neutron imaging is a characterisation tool well suited for mapping the presence of hydrogen in materials. Because of the high attenuation of neutrons with hydrogen, it has been widely used to study hydrogen embrittlement in metals, hydrogen in welds, *etc*.^16^ In this work we look at the distribution of hydrogen in Ti6Al4V–Mg0.6Zn0.5Ca hybrid sample upon corrosion for different durations of time, in 3D using neutron tomography. The chemistry of the Mg part was altered compared to our previous investigation; Zn was added to the alloy composition. Zn is a nutritionally essential element for the human body and is hence safe for bio-medical applications, further adding Zn as an alloying element to Mg is known to slow down its corrosion.^17,18^

The aim of this work is to study 3D hydrogen distribution in microscopical length scale using neutron tomography with special attention to whether the distribution is uniform or limited to the surface. Further phases and microstructural changes upon hydrogen dissolution were also investigated using synchrotron X-ray diffraction (XRD).

## Materials and methods

### Sample preparation

Ti6Al4V–Mg0.6Zn0.5Ca (Ti64–MgZnCa) hybrid samples were prepared by a powder metallurgy route. The different steps are briefly explained below.

### Feedstock preparation

Ti64 powder (spherical, particle size <45 µm) was procured from AP&C, Quebec, Canada. This powder was mixed with 10 wt% binder, which was a mixture of ethylene vinyl acetate (EVA), paraffin wax (PW) and stearic acid (SA). The powder and binder were kneaded in a Z-blade mixer (Femix KM 0.5 K, Linden, Marienheide, Germany) for two hours at a temperature of 120 °C in a glovebox under a controlled argon atmosphere.

For MgZnCa feedstock, alloy powder (spherical, <45 µm particle size) was purchased from SFM, Martigny, Switzerland. 77.3 wt% of this powder blend was mixed with a binder made of PW components, SA and polypropylene. This mixture was then preheated (150 °C) and mixed using a planetary mixer (ARE-250, Thinky Corporation, Tokyo, Japan) in a glovebox under a controlled argon atmosphere. Both Ti64 and MgZnCa feedstock were granulated after cooling.

### Metal injection molding (MIM), de-binding and sintering

Dog-bone shaped samples (ISO 2740-B standard) of Ti64 alloy were prepared using metal injection moulding. The prepared feedstock was fed into an industrial injection moulding machine (320S Allrounder, Arburg, Germany) at an injection pressure of 800 bar, an injection rate of 35 cm^3^ s^−1^, an injection temperature of 112 °C and a mold temperature of 43 °C to form the ‘green parts’.

This was followed by two-step debinding: solvent and thermal. Solvent debinding was carried out by dipping samples in hexane at 40 °C for 15 h in a LÖMI EBA 50 debinding facility (LÖMI, Grossostheim, Germany) to remove the wax and steric acid with hexane as solvent. After this, the remaining binders were removed *via* thermal debinding by heating in a furnace (Nabertherm, Lilienthal, Germany) using 70 L h^−1^ argon flow at 12 mbar and temperature between 250 and 400 °C. This was followed by sintering in vacuum (10^−5^ mbar) at 1300 °C for 2 h.

**Fig. 1:**
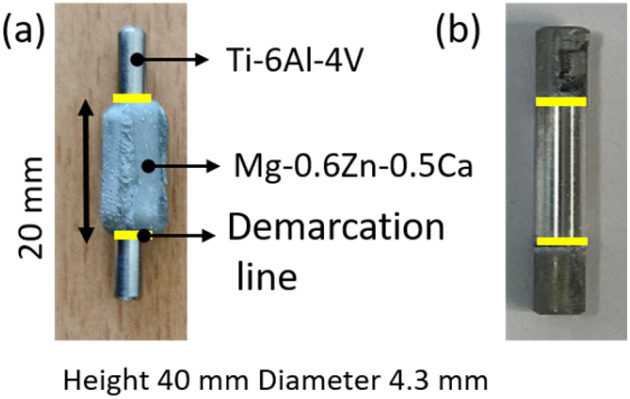
Photo of (a) a hybrid Ti64–MgZnCa sample (b) Sample after corrosion, and removal of any remaining Mg alloy. ‘Demarcation line’ shown in yellow in both figures.

One Ti64 sample was kept as a reference (reference 1 in Table 1), to the remaining sintered Ti64 samples, MgZnCa was added on the center (see Fig. 1a). A special mold and the MgZnCa feedstock were used in a Babyplast injection molding device (Babyplast 6/12, RAMBALDI + CO.I.T. s. r.l. Molteno, Italy).

**Table 1:** Description of different samples measured

Sample name	Description	Time in corrosion medium (hr)
Reference 1	Ti64	0
Reference 2	Ti64–MgZnCa hybrid	0
Sample 1	Ti64–MgZnCa hybrid	3
Sample 2	Ti64–MgZnCa hybrid	6
Sample 3	Ti64–MgZnCa hybrid	12.5
Sample 4	Ti64–MgZnCa hybrid	24
Sample 5	Ti64–MgZnCa hybrid	120 (complete dissolution)

The Ti64–MgZnCa samples obtained were once again subjected to a two-step debinding in hexane followed by thermal debinding by heating in a 50 mbar argon flow at 400–550 °C for 8 h and sintering for 64 h at a temperature of 638 °C in Ar atmosphere.

The heads of the dog-bone tensile samples were cut using a diamond saw in the presence of lubricant to obtain samples of about 40 mm in height, as shown in Fig. 1(b).

### Corrosion of sample

The Ti64–MgZnCa hybrid samples were immersed in an aqueous solution of 0.9 wt% NaCl at room temperature for different durations ranging from 0 to 120 h (see Table 1 for details). For sample 1 to 4 all the MgZnCa could not be removed by corrosion, so the remaining was scraped off using a grinding paper.

### Characterizations

#### Synchrotron X-ray microtomography

Synchrotron X-ray microtomography was performed at the EH4 of HEMS P07 beamline of DESY,^19^ for sample 5 (full degradation).

The photon energy was 80 keV with a field of view of 1.9 × 3 mm^2^ (V × H). The measurements were done using 8 height steps from top to middle and two scans in each height step to extend the field of view (FOV). Later, all steps were stitched. It took for 8000 projections, approximately 6 hours per sample. By this procedure, half of the sample length was covered, which should be sufficient as the sample is expected to be symmetrical *versus* the middle plane. The effective pixel size was 0.411 µm. Before tomographic reconstruction, the different height steps were stitched, followed by the reconstruction based on a Matlab script using a binning factor of six, thus, resulting in a pixel size of 2.469 µm.

#### Neutron tomography

Samples after corrosion were imaged using neutron tomography at the thermal neutron imaging instrument (NEUTRA) at PSI.^20^ A CCD camera (Andor iKon-L) was coupled with 100 mm objective (Zeiss) and 30 µm thick gadolinium oxysulfide scintillator screen (RC-TriTEC & PSI, Switzerland). The acquired images had an isotropic pixel size of 32.6 µm. The exposure time of all the acquired images was equal to 30 s.

A total of six samples were imaged in two tomographic runs. In each tomographic run three samples were placed together on the sample stage and imaged simultaneously. A set of 626 projections was acquired by rotating samples over 360° (the angular step between projections equal to 0.576°). Thirty open beam (radiograph without sample) and 10 dark current images (radiograph acquired without exposure of the neutron beam) were also acquired.

The scattering correction was performed using established black body methodology,^21,22^ for which a set of 26 black-body sample projections evenly distributed over 360° (angular step of 14.4°) and 10 open beam black body images were acquired.

Acquired projections were reconstructed using the ‘Multi projection BP parallel’ with the MuhRec reconstruction tool.^23^

#### Post reconstruction analysis of X-ray tomography

Segmentation to separate the porosities was performed using the ‘Otsu’ threshold and the equivalent diameter of the segmented pores was measured using the ‘measure region props’ using the scikit-image module of python.^24^

#### Post reconstruction analysis of neutron tomography

3D rendering of the reconstructed volume was done using Avizo software. A 2D projection of the 3D volume was obtained by first separating the samples from the background with ImageJ and then for each pixel, a mean of attenuation coefficient of all slices in the direction perpendicular to the long axis (height) was calculated. This was done using the ‘Z-project’ option in ImageJ software (note that the attenuation of the background was not included in this mean).

To obtain the mean neutron attenuation coefficient radially and along the height, first the sample was separated from the background using segmentation, then the centre pixel of the sample was determined. The radii of all pixels in the sample were calculated from this centre, following which the mean of the attenuation coefficient *versus* radius was plotted and averaged along the height.

#### Chemical analysis

Chemical analysis of O, N and H was done using the inert gas fusion technique (ONH836, LECO, St Joseph, MI, USA). Samples were cut in discs (thickness of 1 mm) using a diamond saw in the presence of lubricant to approximated 50 mg pieces for the measurement.

#### Synchrotron X-ray diffraction

Synchrotron X-ray diffraction was performed at the side station of the high energy materials science (HEMS) beamline P07 ^19^ at DESY (Deutsches Elektronen-Synchrotron) in Hamburg using 87 keV X-rays with a spot size of 1 × 1 mm. 30 diffraction patterns were acquired by rotating the sample and this dataset was averaged for analysis. Rietveld refinement, a tool that models a full powder diffraction profile based on crystal structure data, specimen and instrument effects,^25^ was performed using the FullProf software.^26^

#### Scanning electron microscopy

Scanning electron microscopy analysis of the samples was carried out on a ThermoFisher Scientific Quattro S field emission environmental scanning electron microscope (ESEM) operated jointly by the Helmholtz-Zentrum Hereon and Jülich Center for Neutron Science (JCNS). For this purpose, the samples were embedded in epoxy resin and polished to optical grade. In order to prevent the embedded samples from charging and to avoid a disturbing conductive coating, a water vapor partial pressure of 50 Pa was set in the chamber. The SEM micrographs were taken at a working distance of 10 mm with a probe current of 38 nA and an acceleration voltage of 8 kV using Si p–n diode circular backscatter detector (CBS) to enhance material contrast.

## Results and discussions

### X-ray tomography investigation

**Fig. 2:**
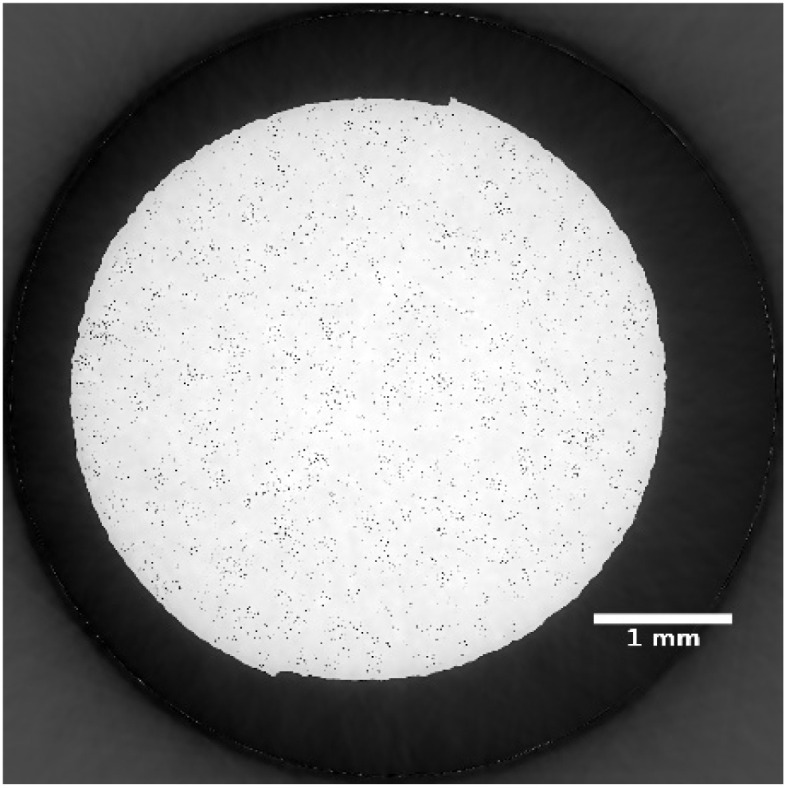
Cross section slice of sample 5 obtained using X-ray microtomography.

Synchrotron X-ray tomography was performed, of a part of the sample with the longest corrosion time (sample 5). A representative cross section slice of the sample is shown in Fig. 2, where no second phase particles or segregation were visible. Porosities were present in the sample and it occupied 0.96 vol%. The pores showed an average equivalent diameter of 10.5 µm and median equivalent diameter of 10.1 µm. The observed porosity can be associated to the manufacturing process, as it is known that sintering produces residual pores depending on powder size distribution, binder and process parameters.^27^

### Neuron tomography and chemical analysis

**Fig. 3:**
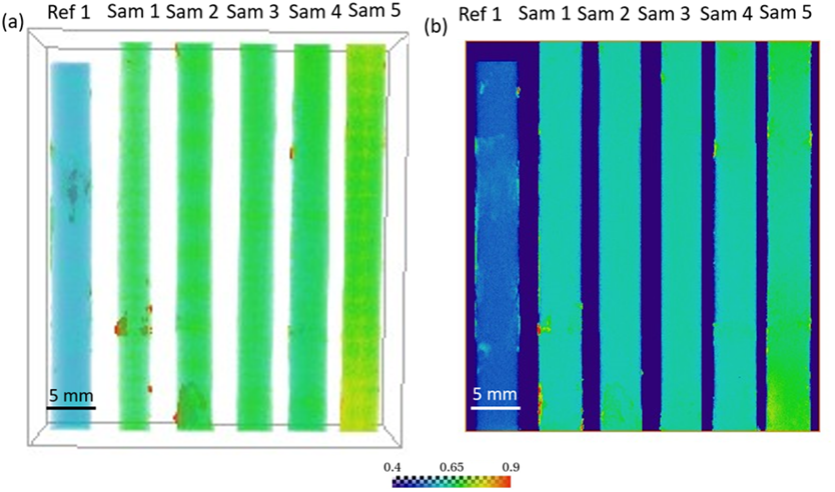
(a) Vertical slice from 3D rendered volume of all the samples (b) 2D projection of mean attenuation coefficient. The colours represent neutron attenuation coefficient value (cm^−1^).

The samples were characterized using 3D neutron tomography to see the effect of dissolution of MgZnCa due to corrosion on Ti64. Fig. 3(a) shows a vertical plane from the 3D rendered volume of all the samples, colour bar represents the neutron attenuation coefficient values in (cm^−1^) and Fig. 3(b) shows a 2D projection of the mean attenuation coefficient. The vertical plane presents the view of a single plane, while the 2D projection is a way to conveniently present an average of the 3D data into a 2D. Samples 1 to 5 did not show any distinguishable feature in the reconstructed data, indicating the absence of any secondary phases, and porosities/defects at this resolution; the only difference observed was in the measured neutron attenuation coefficient value (visible as a change in colours in Fig. 3). From X-ray tomography, we know that the pores are present in a size range that is well below the pixel size of neutron tomography (32.6 µm), therefore, separate pores can not be resolved by the neutron experiment, but their contribution to the neutron attenuation coefficient should be the same for all samples. Upon comparing the neutron attenuation coefficient values in Fig. 3(a) and (b), a consistent increase from sample 1 to 5 was clearly visible while reference 1 showed a lower attenuation than the samples subjected to corrosion. To explain this increase of the attenuation coefficient, we need to consider that hydrogen has a high scattering cross section for thermal neutrons and, hence, even a small amount of hydrogen can be ‘imaged’ using neutron imaging.^28^ Besides, previous studies by our group have already shown the presence of hydrogen in similar samples upon Mg alloy corrosion using chemical analysis.^15^ Hence, this increase of neutron attenuation can be attributed to hydrogen ingress in the samples upon corrosion of the MgZnCa alloy, which increases with the corrosion time. The neutron attenuation coefficient is linked to total neutron cross-section (absorption plus scattering) *via* the number density (*i.e.* number of atoms per unit volume) which can theoretically be used to calculate concentration of hydrogen.^29^ However, the neutron cross sections are energy dependent making these calculations difficult in case of a polychromatic neutron beam.

**Fig. 4:**
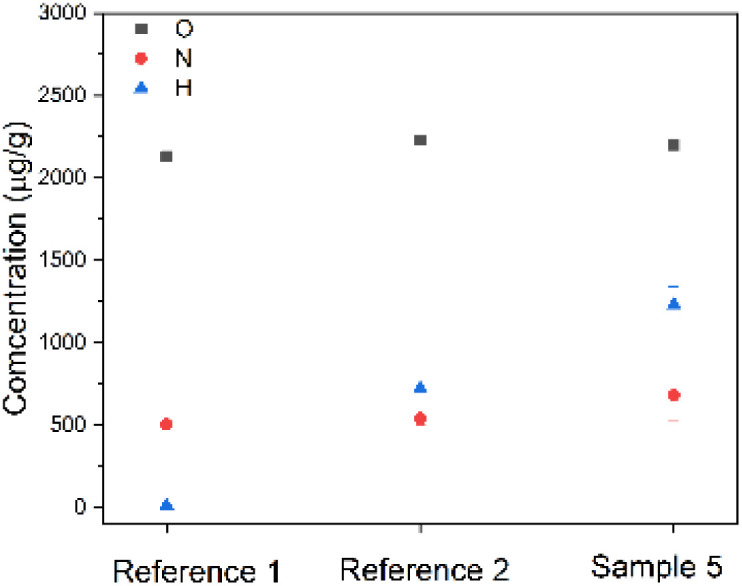
Plot showing chemical analysis of reference 1, reference 2 and sample 5.

To further corroborate and quantify the presence of hydrogen the corroded sample 5 and reference samples 1 and 2 were subjected to chemical analysis, and the results are shown in Fig. 4. All specimens in Fig. 4 show an amount of oxygen of about 2200 µg g^−1^, which is more than the maximum value for titanium grade 5 of 2000 µg g^−1^ (ref. 30) but significantly lower than the critical value of 3200 µg g^−1^, above which severe embrittlement occurs.^31^ According to the phase diagram of titanium and oxygen, the solubility of oxygen in alpha titanium is about 14.5 wt%, which corresponds to 145 000 µg g^−1^.^32^ The formation of TiO_2_ or other forms of titanium oxide phases in the measured specimens can therefore be ruled out. The reference 1 sample did not show any significant presence of hydrogen as expected for the pure alloy. In contrast, sample 5 (hybrid sample with the longest corrosion time) showed over 1200 µg g^−1^ of hydrogen. This confirms that it is the presence of hydrogen in the Ti64 part of the hybrid samples, that causes an increase in neutron attenuation coefficient in sample 5 as compared to reference 1 (Fig. 3).

Ti and its alloys have a good affinity for hydrogen,^33^ but they show a thin oxidation layer on the surface. As the diffusion of hydrogen in TiO_2_ is less than in the pure metal, this oxide layer is known to act as a protective coating against hydrogen absorption in Ti and Ti alloys.^34^ Fast diffusion of hydrogen in bulk Ti and Ti alloys in compassion to TiO_2_ is connected to easy transport of hydrogen along grain boundaries. Hydrogenation of Ti alloy has been reported but at high temperatures and pressure or using electrochemical charging at room temperature. Ti64 alloy can be hydrogenated at room temperature using hydrogen charging, but that requires placing the sample in a suitable electrolyte and applying a certain current density.^35^ Alternatively, subjecting Ti64 alloy to gaseous hydrogen at high temperatures (550–650 °C) and pressure has also shown diffusion of hydrogen into the alloy.^34,36,37^

In the present case, hydrogenation due to corrosion, occurs at room temperature. In the aqueous solutions, water is split electrochemically by Mg and hydrogen gas is formed, which can be absorbed by Ti64. The fact that the hydrogen is being absorbed at room temperature by the Ti alloy indicates that the protective oxide coating on Ti64 is probably compromised. It has been reported that when a slice made of TiO_2_ nanoparticles is compressed with an Mg sheet and infiltrated with an acid at room temperature, there is a reduction of the oxide and oxygen vacancies (*V*_O_) form.^38^ Also, theoretical calculations indicate electron transfer in TiO_2_ interface upon contact with Mg, leading to the reduction of the oxide, and formation of vacancies. Additionally, the excess electrons on the oxide layer promote the formation of hydrogen atoms from hydrogen ion present in the solution, further leading to H absorption into the oxide.^38^

Similarly, in the current situation there are two simultaneous mechanisms at play: the reduction of TiO_2_ protecting layer due to metal reduction reaction, and the corrosion of Mg leading to hydrogen formation. Mg corrosion entails the production of hydrogen gas and can be explained by Volmer–Heyrovsky reaction.^39^ There is electron transfer from Mg (Mg is oxidized to Mg^2+^) into the electrolyte which combines with hydrogen ion present in the saline solution to form hydrogen gas. Simultaneously, because of the higher activity of Mg compared to Ti, there is a reduction of the TiO_2_ coating present on Ti64. Some damage to the TiO_2_ also occurs when Ti64 is sintered with MgZnCa.^15^ Since, the protective coating of Ti64 is compromised this allows for the diffusion of hydrogen into the sample. This has been explained in detail in our previous paper.^15^

Moreover, the chemical analysis (Fig. 4) shows about 500 µg g^−1^ of hydrogen in reference sample 2. This was not expected as during the coating process of Ti64 with MgZnCa the samples were not intentionally subjected to magnesium degradation.

When MgZnCa was added to the Ti64 during sample preparation, the sample was heated up to 638 °C in Argon flow. It has also been reported that prolonged heating at high temperatures (600 °C) in vacuum or inert gas atmosphere can lead to de-hydrogenation.^36,40^ So ideally no hydrogen should be present in the sample. In the present case, however, there was some amount of Mg(OH)_2_ on surface of MgZnCa formed natively under air conditions and there is potential release of hydrogen during thermal debinding of magnesium feedstock and trace amount of hygrogen comes from these sources. Prolonged heating (about 8 h) of the Ti64 sample in hydrogen contained atmosphere is known to cause hydrogenation.^34,36,37^ This is assumed to be the reason why the reference 2 hybrid samples show the presence of some unwanted hydrogen that arises during the sample preparation.

**Fig. 5:**
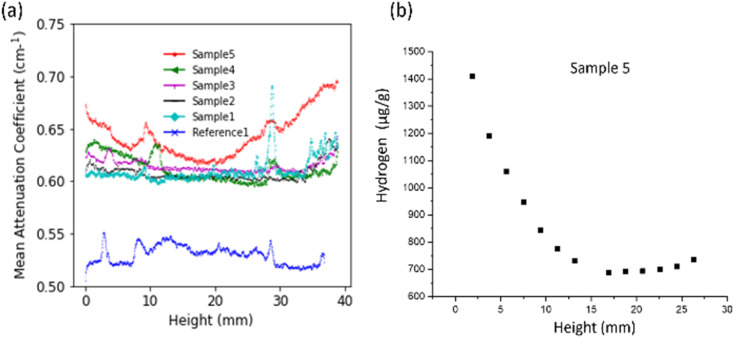
Plot showing mean neutron attenuation obtained from neutron tomography of all samples (a) along height. (b) Concentration sample 5 from chemical analysis.

**Fig. 6:**
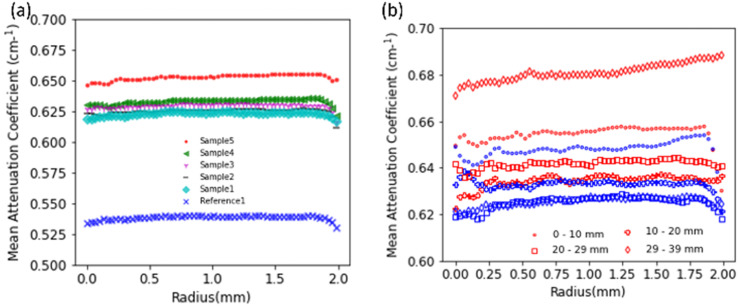
Plot showing mean neutron attenuation obtained from neutron tomography of all samples (a) radially (b) along height (sample 5 in red and reference 1 sample in blue, different markers for different heights are indicated in legend).

To further investigate the distribution of hydrogen inside the samples we look at the mean neutron attenuation coefficient distribution along the height (Fig. 5) and radially (Fig. 6). Fig. 6(a) shows the radial profile for all the samples, where it can be seen that the attenuation remains constant along the radius in all the samples. Further, there is a steady increase in the value between reference 1 and other samples (1 to 5) subjected to corrosion, indicating that as the time spent in corrosive media increases, the attenuation increases. Since longer time in corrosive media corresponds to longer exposure to hydrogen (produced as a consequence on MgZnCa corrosion), this trend further confirms that there is room temperature diffusion of hydrogen into the Ti64 which is causing the increase in neutron attenuation coefficient values steadily between samples. Additionally, we examine the radial profiles of attenuation coefficient at different height ranges (0–10 mm, 10–20 mm, 20–29 mm and 29–39 mm) for sample 5 in comparison to reference 1, where the trends are similar (Fig. 6(b)). While there are variations in value along the height, for each height interval sample 5 always shows a higher attenuation than the reference sample and the radial profile is constant for both samples. This further confirms that along the height of the samples, the hydrogen distributes itself evenly radially.

When looking at the attenuation variation along the height (Fig. 5(a)), the distribution of hydrogen shows some non-uniformity. For example, in sample 5, which has a maximum amount of hydrogen, the edges have more hydrogen compared to centre. Additionally, there is a visible peak in the amount of hydrogen at the demarcation line of regions where MgZnCa was added to the Ti64 alloy (indicated by the yellow line in Fig. 1). The hydrogen concentration obtained from the chemical analysis of a part of sample 5 is shown in Fig. 5(b). A trend similar to the neutron attenuation coefficient values is seen here too, the edges have a higher concentration of hydrogen in comparison to the centre, further reiterating the presence of hydrogen.

The uniformity of the hydrogen distribution hints at the kinetics of the absorption being fast enough to ensure a uniform radial distribution. However, there are certainly some variations seen along the height. Since the regions close to the demarcation line are most prone to damage to the oxide layer, the high hydrogen concentration seen here is understood. A higher hydrogen concentration in the edges compared to the centre is also likely because the edges, which do not have MgZnCa, are exposed to hydrogen for longer durations compared to the centre. However, it was also seen that one edge of the sample showed more hydrogen than the other edge along the height as seen samples 1 and 4 in Fig. 5(a). This is not expected as the samples should be uniform in composition and density. However, it can be seen that the reference 1 sample also shows non uniformity in neutron attenuation value along the height. Since, apart from hydrogen, the chemical composition and density also determines the neutron attenuation, this difference could be attributed to sample non-uniformity during preparation.

### Synchrotron XRD and microscopy

**Fig. 7:**
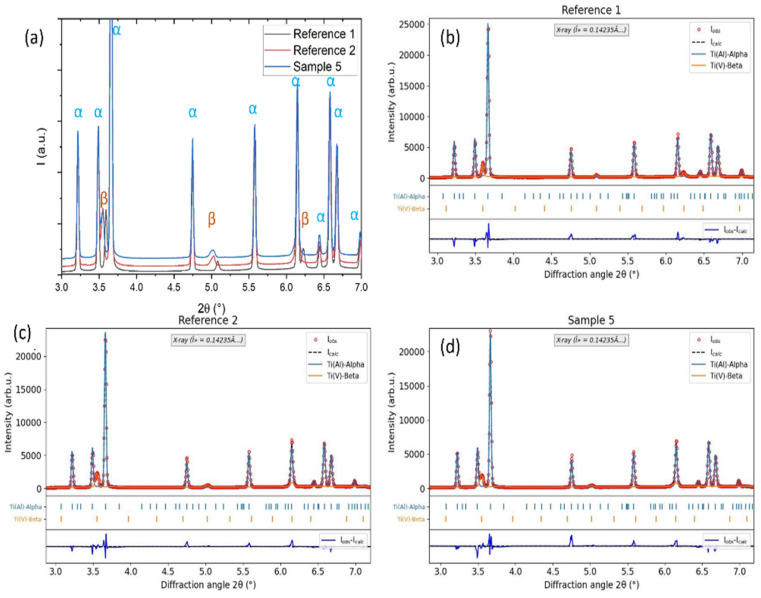
XRD pattern of samples: reference 1, reference 2 and sample 5 (a) and corresponding Rietveld refinements (b–d) by taking into account *α* and *β* phases. The observed data (red circles), calculated data (blue line for *α* phase, orange for *β* phase and black line total fit), and difference plots (blue line), together with the positions of the Bragg reflections, are depicted for all the samples.

To investigate the effect of corrosion and hydrogen ingress on the microstructure of Ti64, we performed synchrotron X-ray diffraction of corroded sample 5 and reference samples 1 and 2. The edges of the reference samples were chosen as the region of interest for diffraction. Fig. 7(a) shows the XRD patterns of the 3 samples (reference 1, reference 2 and sample 5) plotted together for comparison. Fig. 7(b)–(d) depicts the Rietveld refinements of reference 1, reference 2 and sample 5, respectively, by taking into account only Ti α-hexagonal closed packed (HCP) structure (ICSD 76144) with substitutional Al atoms, and Ti β – body-centered cubic (BCC) phase (ICSD 76144) with substitutional V atom for all the samples. No traces of hydrides or oxides were detected and samples consist only of *α* + *β* phases, as expected for Ti64.^41^Table 2 shows the obtained parameters from the Rietveld refinement, *i.e.*, wt% of both phases and corresponding lattice parameters. It can be seen that, in comparison to pure Ti64 (reference 1), there is an increase of *β* phase and a simultaneous reduction of *α* phase in both hybrid samples regardless of the corrosion step (reference 2 without corrosion and Sample 5 after corrosion). It has been widely reported that heating Ti64 alloy in hydrogen atmosphere leads to an increase of *β* phase fraction.^33,37,42^ This happens because hydrogen is a *β* stabiliser, hence, an increase of hydrogen accompanied by high temperature causes the transformation of hydrogenated HCP *α* phase into hydrogenated BCC *β*-phase.^37^ In the present case, when MgZnCa was added to the Ti64 to prepare hybrid samples, there was an additional heat treatment of the Ti64 sample during sintering in an environment containing some trace of hydrogen (from magnesium hydroxide and thermal debinding of feedstock). This led to the *α* → *β* transformation in reference 2 and sample 5. Further, the hydrogenation of sample 5 due to corrosion of MgZnCa happened at room temperature, where hydrogen charging does not induce this phase transformation. Therefore, the *α* and *β* phase fractions are the same in reference 2 and sample 5 within the error bars.

**Table 2:** Phase fraction and lattice parameters of phases obtained by Rietveld refinement

Sample	*α* (wt.%)	*β* (wt. %)	*α* (lattice param *a* = *b*, *c* in Å)	*β* (lattice param *a* = *b* = *c* in Å)
Reference 1	92.1(8)	7.9(2)	2.9226(2), 4.6701(4)	3.2068(6)
Reference 2	89.9(7)	10.1(3)	2.9255(2), 4.6738(3)	3.2497(5)
Sample 5	90.3(7)	9.7(2)	2.9241(2), 4.6717(3)	3.2533(6)

Looking at the lattice cell parameter variations, the lattice parameter for the *α* phase does not show substantial variation for the 3 samples, as can be seen in Fig. 7(a), in which the Bragg peaks corresponding to the *α* phase remain in the same diffraction angle. The diffusivity of hydrogen is less in the close packed HCP crystal structure of the *α* phase, hence, the lattice expansion is not considerable. Moreover the hydrogenated *α* phase is known to get converted to *β* phase as seen before. When we look at the *β* lattice, an expansion in lattice volume by 1.32% is seen in reference 2 in comparison to reference 1. Sample 5 shows a 1.43% increase in lattice volume with respect to reference 1 and a 0.11% increase in lattice volume with respect to reference 2. This is visible in the XRD patterns as relative shifts in the *β* peaks seen in Fig. 7(a). When hydrogen diffuses into the *β* phase, it occupies the interstitial sites of the BCC crystal lattice, which results in the expansion of lattice volume as shown in the following studies^35,43,44^ both for high temperatures hydrogenation and room temperature hydrogen charging. For example, a high temperature charging of 517 µg g^−1^ deuterium into Ti64 with similar microstructure resulted in 0.5% volume expansion of the *β* phase.^44^ In this study, the expansion of *β* phase in sample 5 with respect to reference 2 indicates that the room temperature hydrogen diffusion in Ti64 due to corrosion causes a lattice expansion of the Ti BCC *β*-phase. And the expansion of *β* phase in reference 2 with respect to reference 1 indicates that the unwanted hydrogen diffusion during the sintering of MgZnCa also leads to an expansion of Ti BCC *β*-phase (along with the *α* → *β* transformation).

Further, hydrogen ingress at high temperatures exceeding a certain limit has been known to cause the formation of hydride phases.^35,37,43^ This was also reported in pure Ti based hybrid sample in our previous work,^15^ in the present case, however, no such hydride phases were seen. This can be attributed to the fact that the hydrogen content of the samples is too low to form hydrides which has also been reported previously.^34,44^

**Fig. 8:**
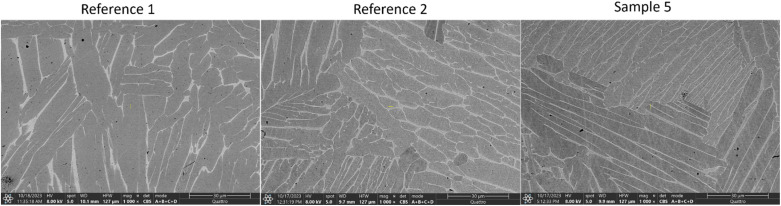
SEM micrographs of reference 1, reference 2 and sample 5.

Lastly, we look at the SEM microstructure of both reference samples without corrosion, pure Ti64 (reference 1), Ti64–MgZnCa hybrid (reference 2) and the hybrid sample subjected to full corrosion (sample 5) presented in Fig. 8. All samples show two phases: a dark *α* phase in the SEM in a predominantly lamellar orientation surrounded by a bright network of *β* phase at the grain boundaries with a thickness of approximately 500 nm. EDX measurements show that the *β* phase is richer in vanadium (atomic ratio: Al : 7, Ti : 81, V : 12) compared to the *α* phase (atomic ratio: Al : 11, Ti : 88, V : 3). Hydrogen is preferentially absorbed by the *β* phase, and since the network of *β* phase is well connected this enables uniform hydrogen distribution in the hydrogenated samples as was seen radially with neutron imaging (Fig. 6(a)). Apart from this, there were no second phase particles visible, and a marked increase in Ti BCC *β*-phase was seen in reference 2 and sample 5 in comparison to reference 1 sample, as also confirmed by XRD.

## Conclusions

Hybrid Ti64–MgZnCa samples were immersed in a 0.9 wt% NaCl aqueous solution, leading to the dissolution of MgZnCa and evolution of hydrogen. This hydrogen diffused into the Ti64 even at room temperature. With neutron tomography a radially uniform distribution of hydrogen was confirmed while along the height, the edges showed more hydrogen than the centre. This can be attributed to the centre being covered with MgZnCa for most of the time during corrosion preventing hydrogen diffusion.

The room temperature hydrogen ingress into Ti64 led to a volumetric expansion of the Ti BCC *β*-phase, while no hydride formation was observed with synchrotron XRD.

When designing hybrid implants, care must be taken to make sure that the hydrogen that evolved from corrosion does not lead to hydrogenation and consequent embrittlement of the permanent implants. This also becomes important if a new temporary implant is added near an existing permanent Ti-based implant.

## Data availability

Data for this article, including neutron tomography are available at zenodo repository at https://doi.org/10.5281/zenodo.14230315.

## Author contributions

RK and VG conceptualisation, investigation and writing original draft. CS, PT, AK, WL, DCFW, JM and MS investigation and formal analysis, CB, TE and RW supervision and resources. All authors took part in reviewing and editing.

## Conflicts of interest

There are no conflicts to declare.
